# Divergence in the tomato rhizosphere microbial community structure driven by three soil types

**DOI:** 10.1128/spectrum.03031-25

**Published:** 2026-03-26

**Authors:** Ken Chen, Xinru Lin, Xiao Wei, Yan Yin, Mingqin Ye, Shangdong Yang

**Affiliations:** 1 Guangxi Key Laboratory of Agro-environment and Agro-product Safety, National Demonstration Center for Experimental Plant Science Education, Agricultural College, Guangxi University 12664, Nanning, China; Nova Southeastern University, Fort Lauderdale, Florida, USA

**Keywords:** tomato (*Solanum lycopersicum *L.), soil types, rhizospheres, soil fertility, soil microbial community structure

## Abstract

Loess, calcareous, and laterite soils are common in Guangxi, southern China, where tomatoes are widely cultivated. To understand how these soil types affect tomato growth and the associated microbial communities, we analyzed soil fertility and the rhizosphere microbiome. Tomatoes in calcareous soil exhibited higher β-glucosidase activity and microbial biomass carbon, whereas those in laterite soil showed increased acid phosphatase activity and microbial biomass phosphorus. Actinobacteriota, Proteobacteria, and Acidobacteriota were the dominant bacterial phyla across all soils, while Ascomycota and Basidiomycota predominated among fungi. Each soil type hosted distinct microbial communities: loess was enriched with the bacterial genera *Ramlibacter* and *Bradyrhizobium* and the fungus *Alternaria*; calcareous soil favored the bacterium *Rubrobacter* and the fungus *Fusarium*, and laterite supported bacteria such as *Bacillus* and *Sphingobium*, along with the fungus *Curvularia*. Given its higher phosphorus availability and the presence of beneficial microbes like *Bacillus*, laterite soil appears to provide a more favorable environment for tomato cultivation compared to loess and calcareous soils.

**IMPORTANCE:**

Soil type is a critical but often overlooked factor influencing tomato productivity in southern China, where diverse soils such as loess, calcareous soil, and laterite are extensively cultivated. Understanding how these soils shape rhizosphere microbial communities and soil nutrient dynamics is essential for improving crop performance. This study provides the first comparative assessment of tomato-associated microbiomes across these major soil types in Guangxi. Our findings reveal that each soil fosters distinct microbial assemblages and enzyme activities, with laterite particularly enriched in beneficial taxa such as Bacillus and associated with enhanced phosphorus availability. These insights highlight the importance of soil-specific microbial processes in supporting tomato growth and offer a scientific basis for selecting and managing soils to optimize productivity. The results also contribute to broader efforts to harness rhizosphere microbiomes for sustainable agricultural improvement.

## INTRODUCTION

Soil quality is a critical determinant of plant growth and productivity, fundamentally influenced by nutrients such as nitrogen (N), phosphorus (P), and potassium (K), along with organic matter (1–7). Among various factors, soil type is a key determinant of soil microbial communities, significantly shaping their species composition, abundance, and structure in the rhizosphere microbiome (8, 9). Soil physicochemical properties, including pH, porosity, salinity, and mineral content, are known to influence microbial diversity and activity, often more strongly than the host plant genotype (10–16). These properties interact with microbial communities most dynamically in the rhizosphere—the soil zone surrounding plant roots, where intense energy and nutrient exchange between plants and microbes occur.

Dubbed the plant’s “second genome,” the rhizosphere plays a pivotal role in nutrient cycling and soil fertility, directly contributing to plant health and growth (17–20). Microorganisms in the rhizosphere accelerate nutrient cycling, enhance biological nitrogen fixation, and bolster plant resistance to disease and abiotic stresses, such as drought and salinity (21–27). Additionally, beneficial microorganisms can modify plant traits, including root architecture and fruit quality, thereby enhancing plant productivity (28–30).

Tomato (*Solanum lycopersicum* L.) is a globally important vegetable crop, valued for its richness in vitamins, lycopene, and minerals (31, 32). However, its production is facing growing challenges from soil-borne diseases and declining soil buffering capacity, which impede plant growth and productivity (33, 34). A deeper understanding of how different soil types shape the tomato rhizosphere microbiome is therefore crucial for developing strategies to improve plant resilience.

Despite their importance, the mechanisms through which soil type influences the microbial composition and function of the tomato rhizosphere microbiome remain poorly characterized. Investigating this relationship is essential for optimizing tomato cultivation and improving agricultural sustainability.

This study was conducted in Guangxi, southern China, a region characterized by complex topography and diverse soil types. We focus on the three most widespread soils in Guangxi—loess, calcareous soil, and laterite—to analyze their effect on rhizosphere fertility and microbial communities in tomato. Our objectives were to elucidate how these soil types influence tomato growth and to explore the underlying mechanisms. The findings are expected to provide a theoretical basis and technical support for the scientific planning of tomato production areas and the efficient cultivation of high-quality tomatoes.

## MATERIALS AND METHODS

### Soil types and experimental design

Three soil types—loess, calcareous soil, and laterite—were collected from Qixing county (110°32′ E, 25°25′ N), Wuming county (108°17′ E, 23°2′ N), and the Xixiangtang district of Nanning city (108°17′ E, 22°50′ N) in Guangxi Province, China. The physicochemical properties of these soils are presented in Table 1.

**TABLE 1:** Soil physicochemical properties of three soil types^*a*^

Soil	pH	SOM (g/kg)	TN (g/kg)	TP (g/kg)	TK (g/kg)	AN (mg/kg)	AP (mg/kg)	AK (mg/kg)
Loess (CK1)	5.34 ± 0.05b	4.54 ± 0.56c	0.22 ± 0.02c	0.42 ± 0.06b	8.61 ± 0.55a	76.33 ± 7.43b	9.06 ± 0.64b	104.33 ± 1.53a
Calcareous soil (CK2)	6.42 ± 0.09a	33.56 ± 0.32a	1.75 ± 0.03a	1.12 ± 0.02a	4.01 ± 0.12b	112.00 ± 6.80a	14.22 ± 1.84a	37.33 ± 0.58b
Laterite (CK3)	4.48 ± 0.04c	7.20 ± 0.59b	0.55 ± 0.02b	0.54 ± 0.01b	8.51 ± 0.53a	88.83 ± 10.78b	8.85 ± 0.94b	103.33 ± 1.54a

^*a*^ All data are presented as mean ± standard deviation. Different letters in the same column indicate significant differences (two-tailed Duncan's test) between treatments at *P* < 0.05. SOM, soil organic matter; TN, total nitrogen; TP, total phosphorus; TK, total potassium; AN, available nitrogen; AP, available phosphorus; AK, available potassium.

Also, the tomato variety, Ounuo (purchased from Beijing Youfeng Agricultural Technology Co., China) was used in this study. Seeds were surface-sterilized and sown in a seedling tray. Seedlings at the five-true-leaf stage (approximately 30 days after sowing) were transplanted into the experimental pots on 15 March 2022 with one seedling per pot.

The pot experiment was conducted in a greenhouse at the vegetable base of the College of Agriculture, Guangxi University (108^◦^17′ E, 22^◦^51′ N). The greenhouse was maintained under the following conditions: a day/night temperature regime of 25°C ± 2°C/18°C ± 2°C, relative humidity of 60%–75%, and reliance on ambient daylight. To ensure that nutrient availability was non-limiting and that observed plant growth differences could be primarily attributed to the soil type treatments, a standardized nutrient solution (half-strength Hoagland’s solution) was applied once a week at a rate of 500 mL per pot throughout the experimental period. Six treatments were established: tomatoes grown in (i) loess, (ii) calcareous soil, and (iii) Laterite, along with corresponding unplanted controls for each soil type: (iv) loess (CK1), (v) calcareous soil (CK2), and (vi) laterite (CK3). Cylindrical pots (25 cm radius, 50 cm height) were used. Each pot was filled with 25 kg of soil, and each treatment consisted of 30 replicate pots. All pots were maintained under identical greenhouse conditions and management practices.

### Soil sampling

Rhizosphere soil samples were randomly collected at tomato maturity using the shaking method (35). Briefly, a shovel was disinfected with a spray of 75% ethanol. It was then used to loosen the soil by digging a depth of 50 cm within a radius of approximately 30 cm around the plant roots. The root systems, along with the adhering soil, were carefully pulled up. After shaking the bulk soil, rhizospheric soils were carefully collected from the roots, placed into sterile plastic bags, and stored on ice in a polystyrene foam box. Composite samples were created by thoroughly mixing soil from three random locations within the rhizosphere zone of each replicate pot. Samples were immediately placed in sterile bags, transported on ice to the laboratory, and stored at −80℃ until processing. In the laboratory, each composite sample was divided into two subsamples: one subsample was sieved through a <2 mm mesh while field-moist and stored at 4℃ for short-term analysis of soil biological properties (within 1 week) or at −80℃ for microbial community structure analysis. The other subsample was air-dried at room temperature and sieved (<0.149 mm mesh) and stored for analysis of soil physicochemical properties.

### Soil physicochemical and biological properties

Soil pH was measured potentiometrically in a 1:2.5 (wt/vol) soil: water suspension using a pH meter; soil organic matter (SOM) content was determined by the potassium dichromate oxidation (Walkley-Black) method with external heating (36); total nitrogen (TN), total phosphorus (TP), and total potassium (TK) contents were determined using the Kjeldahl digestion method, alkali fusion molybdenum blue spectrophotometry method, and alkali fusion-flame followed by flame photometry (37, 38). Available nitrogen (AN), available phosphorus (AP), and available potassium (AK) were determined by the alkali hydrolysis diffusion method, the molybdenum blue spectrophotometry method, and flame photometry (39). Soil β-glucosidase, aminopeptidase, and phosphatase activities were determined by the method of Li Zhengao (40); soil microbial biomass carbon (MBC) content was determined by volumetric analysis (41), microbial biomass nitrogen (MBN) content was determined by the ninhydrin colorimetric method (42), and microbial biomass phosphorus (MBP) content was determined by the phosphomolybdenum blue colorimetric method (43).

### Analysis of soil microbial diversity

The tomato rhizosphere soil and background soil microbial community structure were sent to Shanghai Meiji Biomedical Technology Co. Total DNA was extracted according to the instructions of the E. Z. N. A. Soil DNA kit (Omega, USA), DNA concentration and purity were measured using a NanoDrop2000 spectrophotometer (Thermo, USA), and the extracted genomic DNA was examined by 1% agarose gel electrophoresis. Primers 338F (5′-ACTCCTACGGGAGGCAGCAG-3′) and 806R (5′-GGACTACHVGGGTWTCTAAT-3′) were used for PCR amplification of the 16S rRNA V5-V7 region of the inter-rhizosphere soil bacteria. The primers ITS1F (5′-CTTGGTCATTTAGAGGAAGTAA-3′) and ITS2F (5′-GCTGCGTTCTTCATCATCGATFC-3′) were used to PCR amplify the 18S rRNA ITS region of the inter-rhizosphere soil fungus on an ABI GeneAmp Model 9700. PCR products were recovered by 2% agarose gel electrophoresis, purified using the Axy PrepDNA Gel Recovery Kit (AXYGEN), eluted in Tris-HCl, and quantified using the QuantiFluor-ST Blue Fluorescence Quantification System (Promega). The purified amplicons were used for library construction according to the Illumina MiSeq platform standard operating procedures.

### Data processing

The raw FASTQ files were first demultiplexed using a custom Perl script. Quality control and adapter trimming were then performed with fastp (v0.19.6) under the following stringent parameters: we truncated reads at any sites where the average quality score was below 20 within a 50-bp sliding window and discarded any resulting sequences shorter than 50 bp or containing ambiguous bases. We merged the paired-end reads using FLASH (v1.2.7), requiring a minimum 10 bp overlap and permitting a maximum mismatch ratio of 0.2; non-overlapping read pairs were discarded. Final demultiplexing was achieved by exact barcode matching while allowing up to two nucleotide mismatches in the primer region. We then clustered the high-quality sequences into operational taxonomic units (OTUs) at a 97% similarity threshold using the UPARSE algorithm (v7.1), selecting the most abundant sequence as the representative sequence for each OTU. Finally, we assigned taxonomy to these representative sequences with the RDP Classifier (v2.11) against the SILVA 138 (16S rRNA) and UNITE 8.0 (fungal ITS) databases, applying a confidence cutoff of 0.7.

### Statistical analyses

Experimental data were statistically analyzed using Excel 2019 and SPSS 21.0, and Duncan’s multiple range test was used to compare means. Alpha diversity of bacterial and fungal communities was calculated using Mothur (version v.1.30.2, https://mothur.org/wiki/calculators/, accessed on 9 April 2022). Principal coordinate analysis (PCoA) and partial least squares discriminant analysis (PLS-DA) were used for statistical analysis and mapping using R language (version 3.3.1) tools. For microbial community composition and Venn diagram analysis, OTU tables with 97% similarity were selected and used for statistical and mapping purposes using the R language (version 3.3.1) tool. The Kruskal-Wallis H test method was used for significant difference analysis of soil microorganisms. The environmental factors were screened using variance-inflated factor (VIF) analysis, and the relationship between environmental factors, samples, and flora was analyzed using redundancy analysis (RDA). Using PICRUSt, the Kyoto Encyclopedia of Genes and Genomes (KEGG) data set was used to estimate the functional composition of the bacterial community. Functional prediction of fungal communities using the Fungi Functional Association (FUNGuild) tool. An online data analysis was conducted using the free online platform Majorbio Cloud Platform (https://www.majorbio.com/) from the Majorbio Bio-Pharm Technology Co., Ltd. (Shanghai, China). The data were visualized by ImageGP (https://www.bic.ac.cn/ImageGP, accessed on 9 April 2022).

## RESULTS

### Influence of different soil types on tomato yield and quality

As shown in Table 2, calcareous soil produced the tallest tomato plant but the smallest stem diameter, with both parameters showing significant differences compared to loess and laterite. Plants in loess and laterite exhibited similar plant height and stem diameter. Although the yield was similar in loess and laterite, both yielded significantly more than calcareous soil. Laterite-grown fruits achieved the highest levels of soluble solids and soluble sugar, significantly exceeding those from the other two soil types. Vitamin C content was consistent across three soil types. Overall, soil types significantly affected tomato performance. Based on a comprehensive assessment of yield and quality, laterite was identified as the most suitable soil type for cultivation.

**TABLE 2:** The effects of different soil types on the growth, yield, and quality of tomatoes^*a*^

Soil	Height (cm)	Stem diameter (mm)	Yield (kg/hm^2^)	Soluble solids (%)	Soluble sugar (%)	Vitamin C (%)
Loess	149.8 ± 4.37b	17.11 ± 1.18a	115315.67 ± 1215.89a	5.18 ± 0.23b	3.31 ± 0.08b	22.03 ± 1.53a
Calcareous soil	173.5 ± 7.24a	13.76 ± 0.83b	73314.81 ± 1149.42b	5.38 ± 0.12b	3.42 ± 0.15b	22.67 ± 1.67a
Laterite	153.9 ± 6.04b	17.63 ± 0.88a	120021.24 ± 1040.89a	5.71 ± 0.16a	3.84 ± 0.11a	23.27 ± 1.33a

^*a*^ Data present mean ± standard deviation. Different letters in the same column indicate significant differences (two-tailed Duncan’s test) between treatments at *P* < 0.05.

### Enzymatic activity and microbial biomass in rhizospheres of tomatoes grown in three soil types

As shown in Table 3, β-glucosidase activity differed significantly among soil types, being highest in the rhizosphere of tomatoes grown in Calcareous soil, followed by laterite and loess. Aminopeptidase activity was significantly higher in loess rhizosphere soil compared to calcareous soil and laterite, between which activity did not differ significantly. Acid phosphatase activity also varied significantly, with the highest level observed in laterite rhizosphere soil, followed by calcareous soil and loess.

**TABLE 3:** Soil enzymatic activity and MBC, MBN, and MBP in the tomato rhizospheres under the three soil types^*a*^

Soil	β-Glucosidase (nmol/g/min; 30°C)	Aminopeptidase (nmol/g/min; 30°C)	Acid phosphatase (nmol/g/min; 30°C)	MBC (mg·kg^−1^)	MBN (mg·kg^−1^)	MBP (mg·kg^−1^)
Loess	1.35 ± 0.12c	18.05 ± 1.28a	5.46 ± 0.19c	118.97 ± 10.28c	40.66 ± 3.59a	25.97 ± 2.53b
Calcareous soil	3.28 ± 0.19a	15.33 ± 1.47b	7.16 ± 0.58b	365.17 ± 13.26a	39.95 ± 4.76a	25.77 ± 3.06b
Laterite	1.93 ± 0.13b	15.22 ± 1.19b	9.07 ± 0.60a	212.99 ± 13.59b	42.26 ± 2.86a	35.21 ± 1.39a

^*a*^ All data are presented as mean ± standard deviation. Different letters in the same column indicate significant differences (two-tailed Duncan's test) between treatments at *P* < 0.05.

MBC was highest in calcareous soil rhizosphere, followed by laterite and loess, differing significantly among all three soil types. In contrast, MBN showed no significant differences between the rhizospheres of tomatoes grown in the different soils. MBP content was significantly higher in laterite rhizosphere soil than in calcareous soil or loess; however, MBP levels did not differ significantly between calcareous soil and loess.

Similarly, in Table 4, in the black soils without tomato planting, β-glucosidase activity was highest in calcareous soil, followed by laterite and loess, with significant differences between the three soils. No significant differences in aminopeptidase activity were found between loess, calcareous soil, and laterite. Acid phosphatase activity was also highest in laterite, followed by calcareous soil and loess, with significant differences observed among the three soils.

**TABLE 4:** Soil enzymatic activity and MBC, MBN, and MBP in the three black soils^*a*^

Soil	β-Glucosidase (nmol/g/min; 30°C)	Aminopeptidase (nmol/g/min; 30°C)	Acid phosphatase (nmol/g/min; 30°C)	MBC (mg·kg^−1^)	MBN (mg·kg^−1^)	MBP (mg·kg^−1^)
Loess (CK1)	1.13 ± 0.08c	11.32 ± 1.83a	4.71 ± 0.15c	113.49 ± 2.64b	34.86 ± 2.46a	19.57 ± 1.03b
Calcareous soil (CK2)	2.67 ± 0.16a	12.27 ± 1.09a	5.99 ± 0.57b	267.51 ± 25.48a	31.40 ± 1.81a	18.09 ± 0.33b
Laterite (CK3)	1.42 ± 0.11b	10.97 ± 1.28a	6.20 ± 0.18a	120.19 ± 5.72b	35.06 ± 2.25a	26.69 ± 1.62a

^*a*^ Data present mean ± standard deviation. Different letters in the same column indicate significant differences (two-tailed Duncan’s test) between treatments at *P* < 0.05.

MBC was highest in the rhizosphere soil of tomatoes grown in calcareous soil, followed by those grown in laterite and loess, with significant differences between the three soils. MBN showed no significant differences in the rhizosphere soil of tomatoes grown in the three soil types. MBP was highest in the rhizosphere soil of tomatoes grown in laterite, significantly higher than that grown in calcareous soil and loess, but no significant differences were found between tomatoes grown in calcareous soil and loess.

### Alpha diversities of tomato rhizosphere microbes grown in three soil types

The Shannon and Ace indices revealed the diversity and richness of the tomato rhizosphere bacterial communities across different soil types, where higher values indicate greater diversity and richness. As shown in Table 5, sequencing coverage exceeded 98%, indicating credible sequencing depth. Bacterial diversity and richness in rhizospheres of tomatoes differed from soil types. Both measures were highest in laterite, followed by calcareous soil and loess. This pattern was mirrored in the corresponding unplanted soil (CK), confirming soil type as the primary driver of bacterial diversity and richness.

**TABLE 5:** Indicators of soil bacterial and fungal diversity in rhizospheres of tomatoes grown in three soil types^*a*^

Classification	Treatments	Shannon	Ace	Chao1	Coverage
Rhizosphere soil bacteria	Loess	4.98 ± 0.08c	1542.75 ± 105.13c	1499.17 ± 32.33c	0.99
	CK1	4.44 ± 0.05c	881.81 ± 135.40c	886.27 ± 133.28c	0.99
	Calcareous soil	5.97 ± 0.02b	2334.50 ± 109.43b	2320.10 ± 109.66b	0.98
	CK2	5.65 ± 0.15b	1951.73 ± 114.53b	1957.90 ± 124.99b	0.98
	Laterite	6.51 ± 0.04a	2815.10 ± 27.43a	2812.31 ± 36.43a	0.98
	CK3	6.40 ± 0.05a	2630.98 ± 20.80a	2634.21 ± 29.39a	0.98
Rhizosphere soil fungi	Loess	3.07 ± 0.14b	357.75 ± 50.18b	355.08 ± 52.20b	0.99
	CK1	2.48 ± 0.26b	98.41 ± 18.34c	97.83 ± 18.35c	0.99
	Calcareous soil	3.34 ± 0.29b	308.20 ± 29.41b	309.51 ± 27.86b	0.99
	CK2	4.08 ± 0.10a	293.24 ± 32.05b	294.53 ± 31.89b	0.99
	Laterite	4.11 ± 0.11a	575.39 ± 15.76a	574.18 ± 14.44a	0.99
	CK3	4.24 ± 0.19a	555.86 ± 8.36a	551.57 ± 2.93a	0.99

^*a*^ Data present mean ± standard deviation. Different letters in the same column indicate significant differences (two-tailed Duncan’s test) between treatments at *P* < 0.05.

Similarly, fungal diversity and richness were also significantly influenced by soil type. The highest values occurred in rhizospheres of tomatoes grown in laterite and their corresponding unplanted control, significantly exceeding those in loess and calcareous soil. Fungal diversity and richness did not differ significantly between the loess and calcareous soil rhizosphere.

Additionally, the influence of soil type was also apparent in the background soils, where the Shannon and Ace indices were highest in laterite (CK3), followed by calcareous soil (CK2) and loess (CK1). This result confirms that soil type significantly alters bacterial diversity and richness, with laterite consistently demonstrating the highest values.

### Beta diversities of tomato rhizosphere microbes grown in three soil types

**Fig 1:**
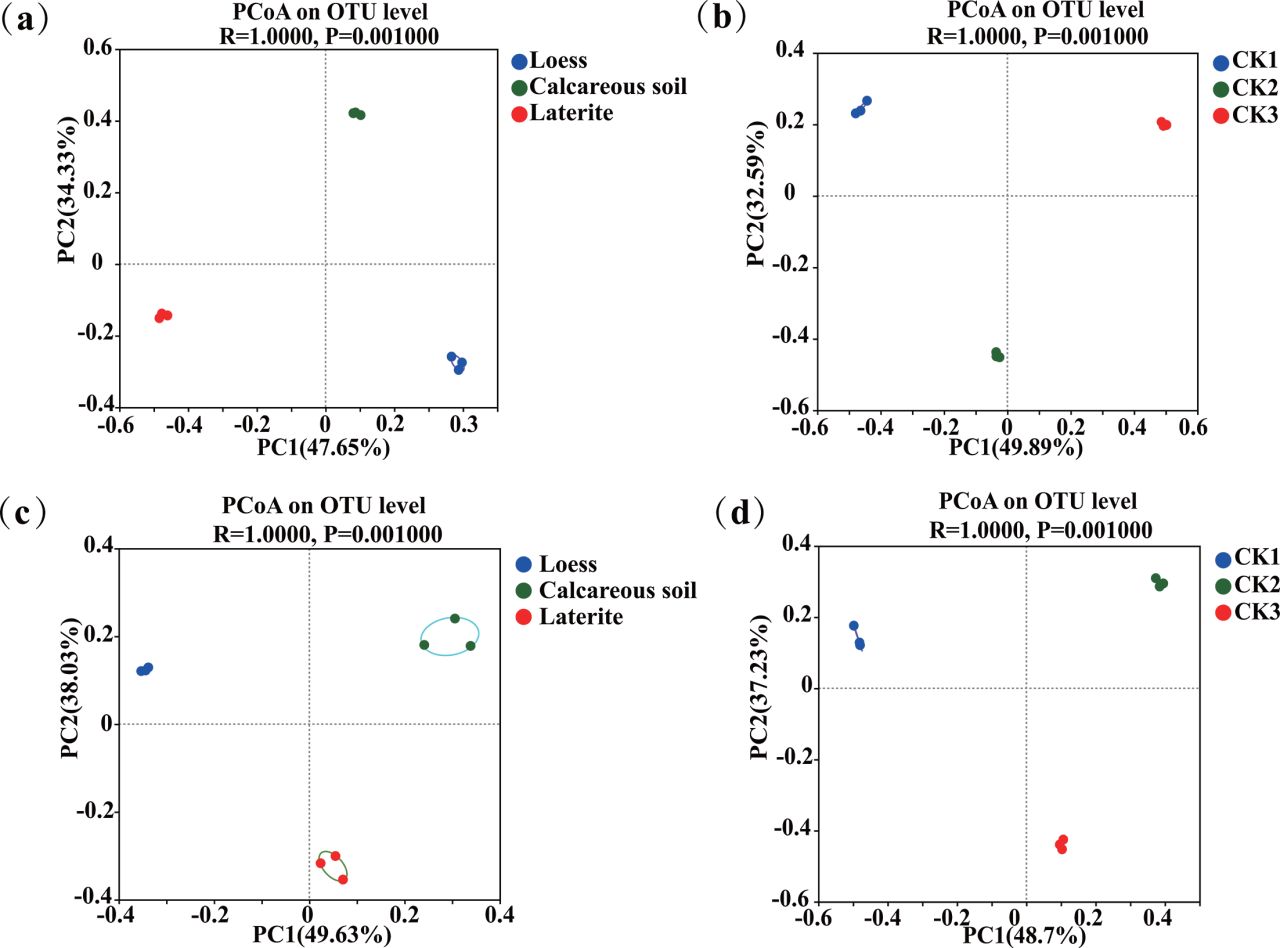
(**a**) Comparison of soil bacterial community structures in rhizospheres of tomatoes grown in three soil types; (**b**) comparison of bacterial community structure in background soil; (**c**) comparison of soil fungal community structures in rhizospheres of tomatoes grown in three soil types; and (**d**) comparison of fungal community structure in background soil.

PCoA of soil bacterial communities at the OTU level revealed significant separation of tomato rhizosphere soil bacterial communities across three soil types (*R* = 1.000, *P* = 0.0010; Fig. 1a), indicating that soil type strongly influenced rhizosphere microbiome assembly. Notably, soil bacterial communities in backgrounds (CK1, CK2, and CK3) exhibited similar trends to those of rhizospheres from the same soil type (Fig. 1b).

Similarly, PCoA of fungal communities (*R* = 1.00, *P* = 0.0010) also showed distinct clustering by soil type for both rhizosphere and backgrounds (CK1, CK2, and CK3) soil samples (Fig. 1c and d), further supporting the dominant role of soil type in shaping microbial assemblages.

### Venn analysis of tomatoes’ rhizosphere microbes grown in three soil types

**Fig 2:**
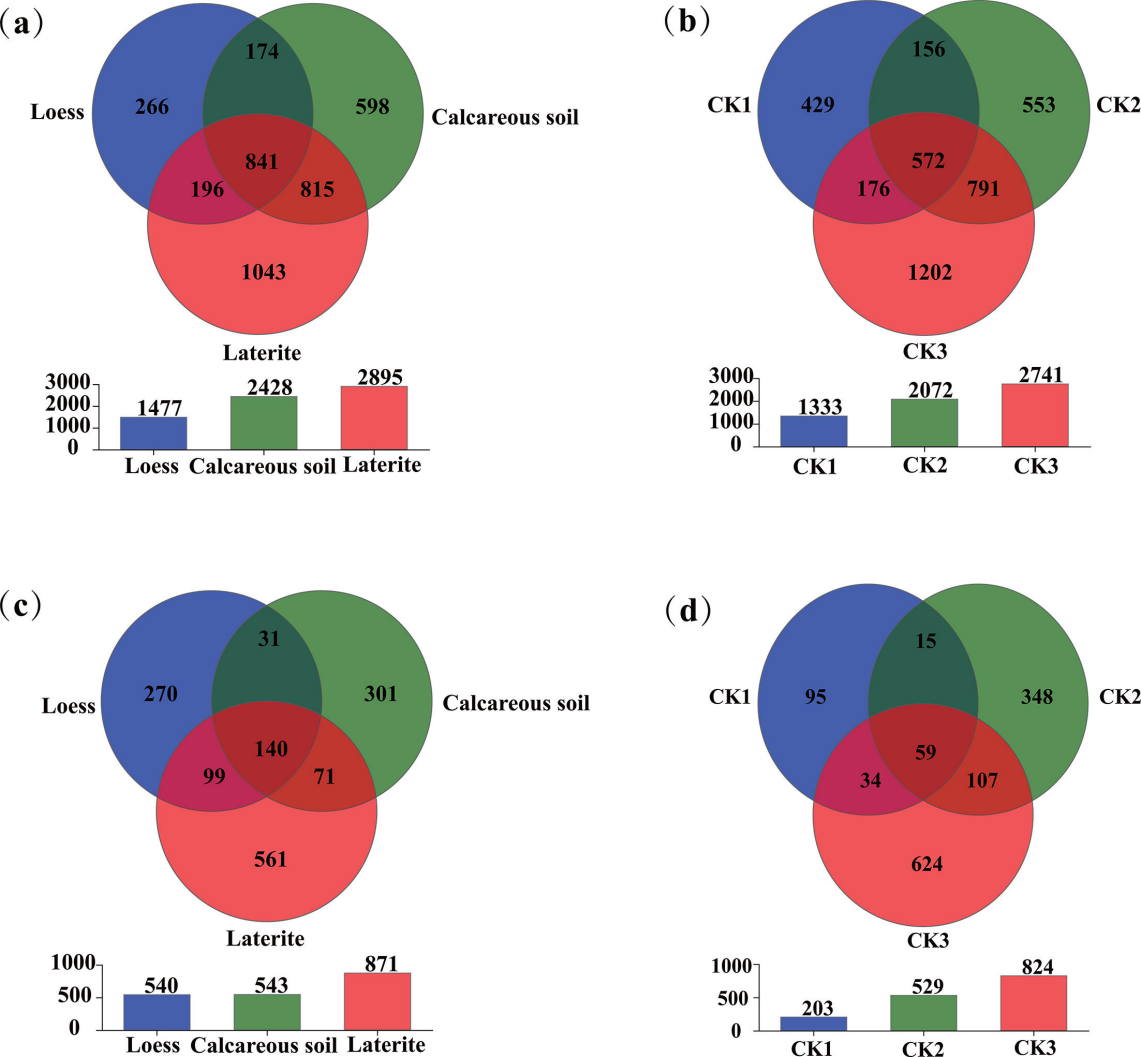
Venn diagrams of bacterial (**a and b**) and fungal (**c and d**) at the OTU level. Diagrams **a** and **c** represent the tomato rhizosphere soils, while diagrams **b** and **d** represent the corresponding background soils, across three soil types.

At the OTU level, the number of unique soil-dominant bacterial OTUs in the tomato rhizospheres varied with soil type: 226 in loess, 598 in calcareous soil, and 1,043 in laterite (Fig. 2a and b). Their corresponding background soils (CK1, CK2, and CK3) contained 429, 553, and 1,202 special dominant bacterial OTUs, respectively. A similar pattern was observed for fungi (Fig. 2c and d). The unique soil-dominant fungal OTUs numbered 44 (loess), 72 (calcareous soil), and 133 (laterite) in the rhizosphere, compared to 95, 348, and 624 in the corresponding background soils. Notably, the background soils consistently exhibited a higher number of unique dominant OTUs for both bacteria and fungi across all soil types, except for bacteria in calcareous soil, where the rhizosphere count was slightly higher (598 vs. 553)

### Soil bacterial and fungal compositions in rhizospheres of tomatoes grown in three soil types

**Fig 3:**
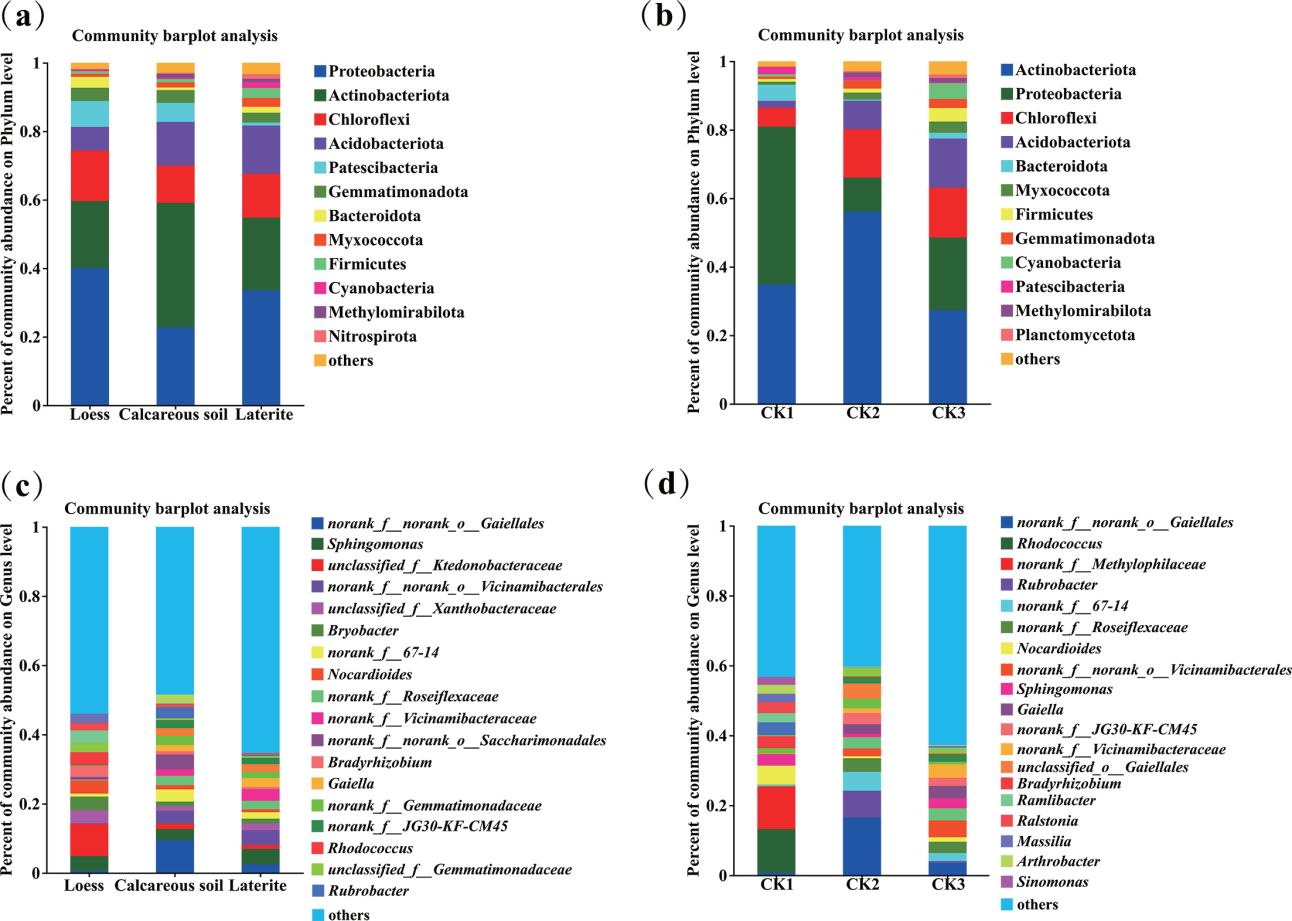
Soil bacterial community composition of tomato rhizosphere in three soil types at the phylum (**a**) and genus (**c**) levels; soil bacterial compositions in the background soil at the phylum (**b**) and genus (**d**) levels.

As shown in Fig. 3a and b, the numbers of soil dominant bacterial phyla (relative abundances are greater than 1%) in rhizospheres of tomatoes grown in loess, calcareous soil, laterite, and their backgrounds (CK1, CK2, and CK3) were 7, 6, 8, and 8, 11, 11, respectively.

Additionally, the unique soil-dominant bacterial phyla in each soil type were as follows: Patescibacteria (2.03%) was in loess (CK1); Cyanobacteria (4.59%), and Planctomycetota (1.06%) in laterite (CK3). In contrast, Firmicutes (2.90%), Cyanobacteria (1.72%), and Nitrospirota (1.33%) were identified as unique phyla in rhizospheres of tomatoes grown in laterite.

At the genus level, the numbers of dominant soil bacterial genera (relative abundance is greater than 1%) in rhizospheres of tomatoes grown in loess, calcareous soil, laterite, and their corresponding backgrounds (CK1, CK2, and CK3) were 25, 25, 24 and 17, 20, 25, respectively (Fig. 3c and d).

Furthermore, distinct bacterial genera were identified as unique to each soil type. The loess soil (CK1) harbored genera such as *Ramlibacter*, *Bradyrhizobium, Ralstonia, Massilia, Sinomonas, Flavisolibacter,* unclassified_ f_ *Micrococcaceae*, unclassified f__ *Oxalobacteraceae*, *Asinibacterium, Rubrobacter*, and norank_ f_ *Methylophilaceae*. In contrast, the calcareous soil (CK2) was characterized by the presence of *Rubrobacter,* unclassified_ O_*Gaiellales*, norank_f_norank_ 0_*Robubacetrals*, norank_f_norank_o_*Microtrichales*, and *Micromonospora*. Finally, the laterite soil (CK3) featured unique genera including *Bacillus*, norank_f_A4b, *Sphingobium,* and *Microvirga*.

Additionally, Ramlibacter, Bradyrhizobium, Ralstonia, Massilia, Methylobacterium-Methylorubrum, norank_ f_ Methylophilaceae, Sinomonas, TM7a, Acidibacter, unclassified_0_Saccharimonadales, Burkholderia-Caballeronia-Paraburkholderia, and Micropepsis were the unique bacterial genera in rhizospheres of tomatoes grown in loess. Also, Rubrobacter, unclassified_ O_Gaiellales, and norank_ f_norank_o_Saccharimonadales were the unique bacterial genera in rhizospheres of tomatoes grown in calcareous soil; Bacillus, norank_f_A4b, Sphingobium, Nitrospira, Pedomicrobium, Microvirga, and Allorhizobium-Neorhizobium-Pararizopbium-Rhizobium were the unique bacterial genera in rhizospheres of tomatoes grown in laterite.

Meanwhile, we also found that *Bryobacte* only significantly enriched in rhizospheres of tomatoes grown in loess, but *Rhodococcus*, *Nocardioides, Bradyrhizobium, Ramlibacter, Ralstonia,* and *Massilia* significantly enriched in rhizospheres of tomatoes grown in loess and its corresponding background (CK1). Moreover, *Rubrobacter was* significantly enriched in rhizospheres of tomatoes grown in calcareous soil and its corresponding background (CK2), and *norank_ f_norank_o_Gaiellale*s was only significantly enriched in calcareous soil background (CK2) (*P* < 0.05, below). Furthermore, *Sphingomonas* and *Lysobacter* were significantly enriched in rhizospheres of tomatoes grown in laterite, and *Bacillus* was only significantly enriched in the laterite background (CK3) (Fig. 3).

**Fig 4:**
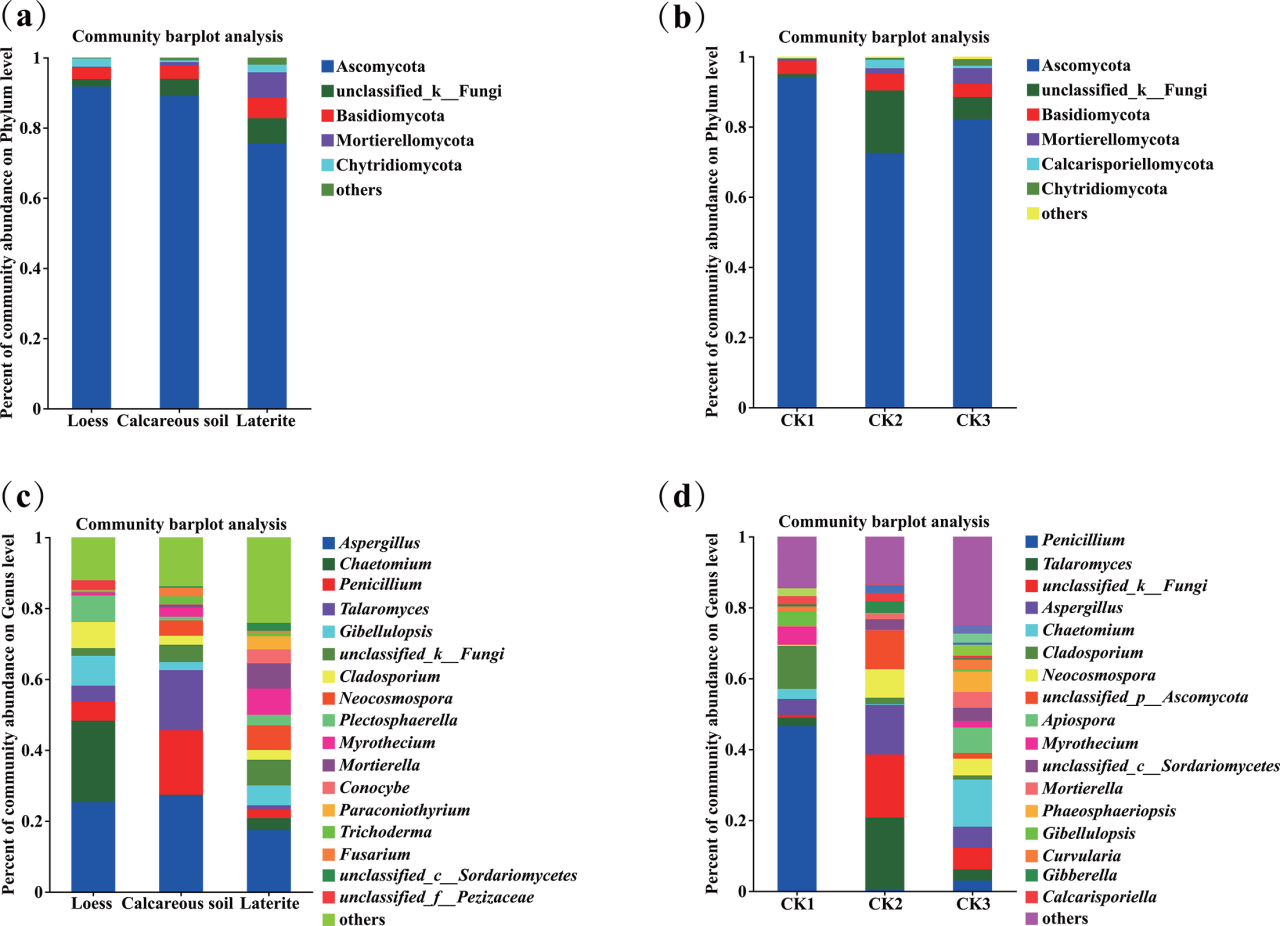
Soil fungal community composition of tomato rhizosphere across three soil types at the phylum (**a**) and genus (**c**) levels; soil fungal compositions in the background soil at the phylum (**b**) and genus (**d**) levels.

As shown in Fig. 4, the numbers of dominant soil fungal phyla (relative abundances greater than 1%) in rhizospheres of tomatoes grown in loess, calcareous soil, laterite, and their corresponding backgrounds (CK1, CK2, and CK3) were 4, 3, 5, and 2, 5, 5, respectively (Fig. 4a and b).

The unique dominant soil fungal phyla differed by habitat: Calcarisporiellomycota (2.42%) was unique to calcareous soil (CK2), Chytridiomycota (1.91%) to laterite (CK3), and Mortierellomycota (7.10%) was uniquely dominant in the rhizospheres of tomatoes grown in both soils.

At the genus level, the numbers of dominant soil fungal genera (relative abundance greater than 1%) in rhizospheres of tomatoes grown in loess, calcareous soil, laterite, and their corresponding backgrounds (CK1, CK2, and CK3) were 11, 14, 21, and 14, 11, 21, respectively (Fig. 4c and d).

Furthermore, the composition of unique fungal genera varied across the soil types. In loess soils (CK1), the unique genera included *Alternaria, Myrothecium, Curvularia*, *Stemphylium, Saccharomyces, Monascus, Trichosporon*, and *Microdochium*. In calcareous soil (CK2), the unique genera were *Neocosmospora*, unclassified_ c_ *Eurotiomycetes*, *Mortierella*, unclassified_ C_ *Sordariomycetes*, *Gibberella*, and *Calcarisporiella*. In laterite (CK3), the unique genera comprised *Curvularia*, unclassified p_ *Chytridiomycota*, *Paraconiothyrium*, *Conocybe*, *Humicola, Apiospora, Phaeosphaeriopsis, Myrmecridium*, and *Albifimbria*.

By contrast, *unclassified_p_ Chytridiomycota*, *Alternaria*, and *unclassified_f_Pezizaceae* were the unique bacterial genera in rhizospheres of tomatoes grown in loess; *Myrothecium*, *Fusarium*, *Rhodctorula*, and *unclassified_ c_ Eurotiomycetes* were the unique bacterial genera in rhizospheres of tomatoes grown in calcareous soil; *Curvularia*, *Mortierella*, *unclassified_ C_ Sordariomycetes*, *Paraconiothyrium*, *Conocybe*, *Stemphylium*, *Humicola*, *Roussoella*, *unclassified_ C_ Chytridiomycetes*, and *unclassified_ c_ Eurotiomycetes* were the unique bacterial genera in rhizospheres of tomatoes grown in laterite.

All the above results revealed that *Chaetomium* and *Gibellulopsis* were significantly enriched in rhizospheres of tomatoes grown in loess, even though *Penicillium* and *Cladosporium* were significantly enriched in their corresponding background (CK1). Meanwhile, *Aspergillus* significantly enriched in rhizospheres of tomatoes grown in calcareous soil, although *Talaromyces*, *unclassified_k_Fungi, Neocosmospora,* and *unclassified_p_Ascomycota* were significantly enriched in their corresponding background (CK2). Moreover, *Mortierella was* significantly enriched in the rhizospheres of tomatoes grown in laterite (Fig. 4).

### RDA of soil bacterial and fungal communities in tomato rhizospheres across three soil types

**Fig 5:**
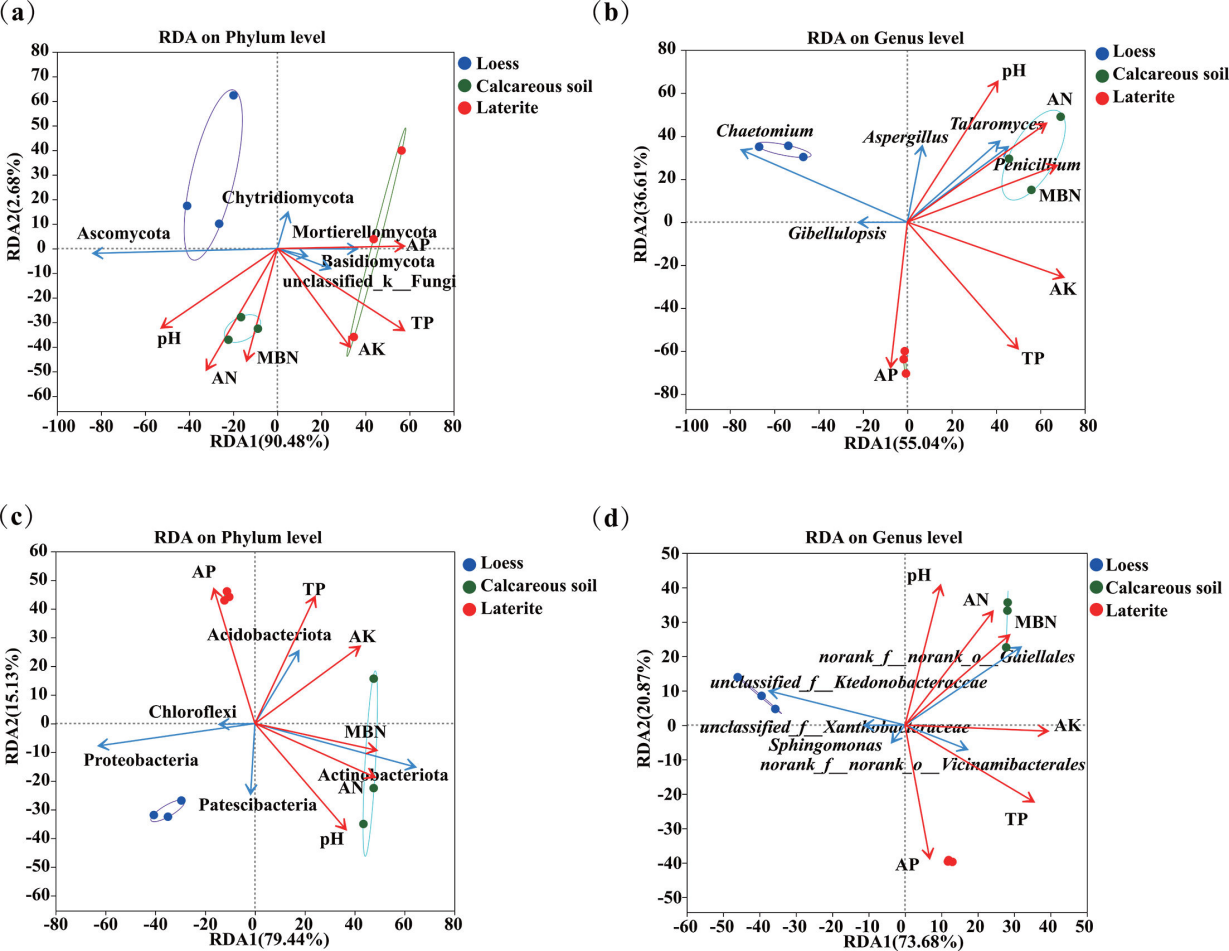
RDA at the phylum level and genus level of soil bacteria (**a and b**) and fungal (**c and d**) communities in the rhizosphere of tomatoes grown in three soil types.

RDA was performed after removing environmental factors with VIF > 10 (organic matter [SOM], TN, TK, β-glucosidase, aminopeptidase, acid phosphatase, MBC, and MBP). As the soil bacterial community at the phylum level, the overall explanation of the bacterial phylum community was 93.16% in the tomato rhizosphere, including 90.48% on the first axis and 2.68% on the second axis. Significant factors: AK (*R*^2^ = 0.556, *P* = 0.003), pH (*R*^2^ = 0.489, *P* = 0.007), and MBN (*R*^2^ = 0.361, *P* = 0.031). Among them, Actinobacteriota was positively correlated with soil pH, MBN, AK, and AN and negatively correlated with TP and AP. Acidobacteriota was positively correlated with TP, AK, and AP and negatively correlated with soil pH (Fig. 5a). Notably, AK was the primary diver of bacterial community composition (*R*² = 0.556, *P* = 0.003), potentially by selecting for potassium-solubilizing taxa like Actinobacteriota, with which it was positively correlated. Soil pH (*R*² = 0.489, *P* = 0.007) and MBN (*R*² = 0.361, *P* = 0.031) were also significant factors, likely through their roles in modulating nutrient availability and habitat conditions.

At the genus level, total constrained variation was 1.65% (RDA1: 55.04%; RDA2: 36.61%). Significant factors were pH (*R*^2^ = 0.511, *P* = 0.005), MBN (*R*^2^ = 0.495, *P* = 0.006), AK (*R*^2^ = 0.453, *P* = 0.009), TP (*R*^2^ = 0.392, *P* = 0.036), and AN (*R*^2^ = 0.322, *P* = 0.05). Meanwhile, *Sphingomonas* were positively correlated with AP and showed a negative correlation with soil pH, TP, MBN, AK, and AN (Fig. 5b).

Soil fungal community at the phylum level, total constrained variation was 94.57% (RDA1: 79.44%, RDA2: 15.13%). Significant factors were AK (*R*^2^ = 0.715, *P* = 0.001), pH (R^2^ = 0.681, *P* = 0.001), and TP (*R*^2^ = 0.390, *P* = 0.018). Among them, Ascomycota was positively correlated with soil pH and AK; Basidiomycota, Chytridiomycota, and Mortierellomycota were positively correlated with AK and TP and negatively correlated with pH (Fig. 5c).

At the genus level, total constrained variation was 65.09% (RDA1: 45.69%, RDA2: 19.40%). Significant factors were pH (*R*^2^ = 0.552, *P* = 0.004), TP (*R*^2^ = 0.486, *P* = 0.007), MBN (*R*^2^ = 0.405, *P* = 0.021), AK (*R*^2^ = 0.356, *P* = 0.033), and AN (*R*^2^ = 0.333, *P* = 0.047). *Aspergillus*, *Talaromyces*, and *Penicillium* were positively correlated with pH, MBN, and AN and negatively associated with AK (Fig. 5d).

In contrast, the fungal community structure was predominantly governed by a different set of factors: pH, AK, and TP. Among these, AK again demonstrated the strongest explanatory power (*R*² = 0.715, *P* = 0.001), followed closely by pH (*R*² = 0.681, *P* = 0.001). The significant influence of TP (*R*² = 0.390, *P* = 0.018) highlights a key distinction from the bacterial community, suggesting fungi are more sensitive to the TP pool. This may be attributed to the high phosphorus demand fungal phyla like Basidiomycota and Mortierellomycota.

All the above results indicated that pH, AK, and MBN were primary drivers of soil bacterial community variation, while pH, AK, and TP dominated fungal community dynamics.

### Functional analysis of soil bacterial communities in rhizospheres of tomatoes grown in three soil types and their corresponding background soil

**Fig 6:**
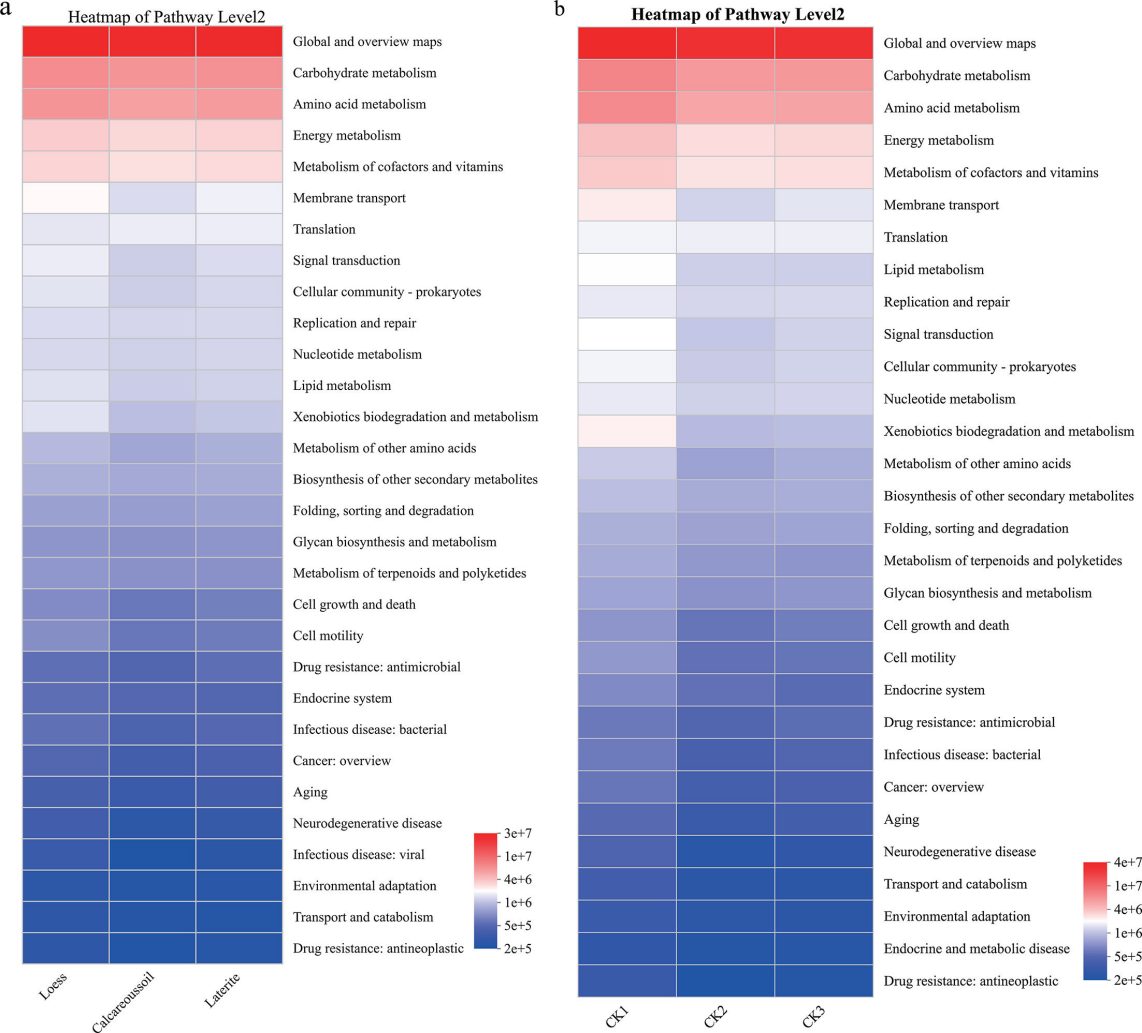
Functional analysis of soil bacteria in rhizospheres of tomatoes grown in three soil types (**b**) and their corresponding background soils (**a**) at hierarchy two levels.

Bacterial metabolic potential in tomato rhizospheres and their corresponding background soils across three soil types (loess, calcareous, and laterite) was predicted using PICRUSt2 with the KEGG database. Six primary metabolic pathways could be found; that is, calcareous soil systems (rhizosphere + its corresponding background soil) exhibited significantly higher metabolism pathway abundance than loess/laterite systems (*P* < 0.05, ANOVA; Fig. 6a). Loess and laterite systems showed significantly higher abundance in Genetic Information Processing, Environmental Information Processing, Cellular Processes, Human Diseases, and Organismal Systems (Fig. 6).

Meanwhile, three core pathways accounted for >56% total abundance: Global and overview maps (39.72%–41.33%), Carbohydrate metabolism (8.9%–9.40%), and Amino acid metabolism (8.04%–8.14%). Calcareous soil upregulated Ccarbohydrate metabolism, and loess upregulated Amino acid metabolism (Fig. 6).

**Fig 7:**
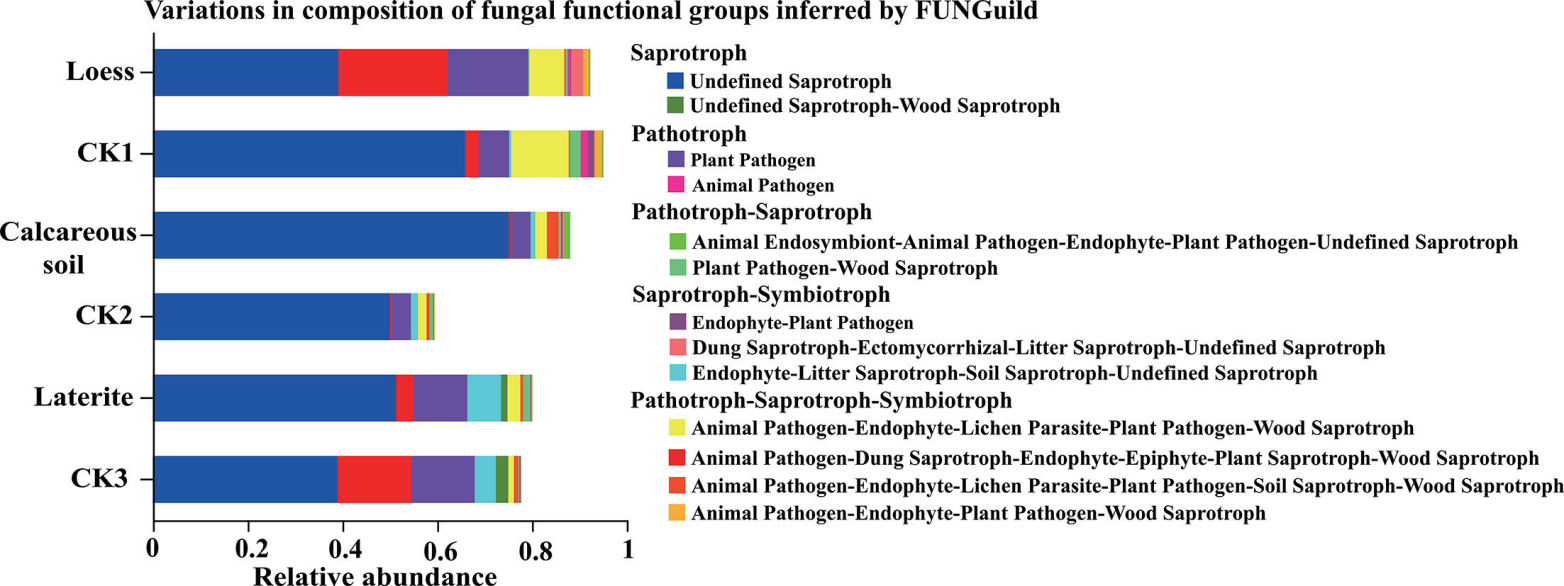
Functional analysis of soil fungal communities in rhizospheres of tomatoes grown in three soil types and their corresponding background soils.

FUNGuild (Fungi Functional Guild) is a tool used for classifying and analyzing fungal communities based on microbial ecological guilds. FUNGuild analysis classified the fungal communities in tomato rhizosphere soil across the three soil types and their background soil into five primary trophic modes: saprotroph, pathotroph, saprotroph-symbiotroph, pathotroph-saprotroph, and pathotroph-symbiotroph (Fig. 7). Significant differences in guild abundance were observed among soil types:

Saprotroph abundance was significantly higher in calcareous soil than those of loess and laterite. Pathotroph and saprotroph-symbiotroph abundances were significantly higher in loess than those of calcareous soil and laterite. Pathotroph-saprotroph and pathotroph-symbiotroph abundances were significantly higher in laterite than those of calcareous soil and loess. These trophic modes were further classified into 13 ecological functional groups (relative abundance is greater than 1%) based on fungal resource acquisition strategies. The dominant functional groups across three soil types were as follows: yndefined saprotroph (relative abundance range: 38.94%–75.16%), plant pathogen (4.10%–17.02%), and AELW/PW (1.82%–12.15%). In loess soil, the relative abundance of plant pathogen and animal pathogen-endophyte-lichen parasite-plant pathogen-wood saprotroph increased significantly compared to the other soil types. In the calcareous soil, the relative abundance of undefined saprotroph and animal pathogen-endophyte-lichen parasite-plant pathogen-soil saprotroph-wood saprotroph increased significantly. In laterite soil, the relative abundance of endophyte-litter saprotroph-soil saprotroph-undefined saprotroph and plant pathogen-wood saprotroph increased significantly (Fig. 7).

## DISCUSSION

### Effect of three soil types on bacterial enzyme activity and microbial biomass in the tomato rhizosphere

Soil enzymes are crucial for maintaining soil biological activity and health (44) directly participating in nutrient cycling processes. This includes the degradation of organic matter and biogeochemical cycles of key elements like nitrogen, phosphorus, and sulfur (45, 46). Consequently, their activity levels serve as sensitive indicators of soil biological activity and quality (47). Soil microbial biomass, another vital component for soil fertility and crop productivity, plays a central role in nutrient cycling and transformation (48). Specifically, MBC reflects soil organic carbon pools (49), while MBN participates in nitrogen mineralization and fixation, and MBP regulates soil phosphorus availability (50).

This study measured key enzymes and microbial biomass components in tomato rhizosphere soil across three soil types (calcareous soil, loess, and laterite). Our findings revealed significant soil-type effects: Calcareous soil cultivation resulted in significantly higher β-glucosidase activity (involved in cellulose degradation and carbon cycling) and MBC component compared to the other soil types; laterite soil cultivation resulted in significantly higher acid phosphatase activity and MBP content compared to the other soil types.

### Characteristics of soil bacterial community structures and their functions in rhizospheres of tomato grown in three soil types

Changes in soil microbial activity and community structure sensitively reflect soil ecosystem quality and health (51) and are closely linked to soil nutrient status, microbial diversity, and richness (52). Furthermore, soil biodiversity influences plant nutrient storage, cycling, and productivity (53). We found that after planting tomato, bacterial diversity and abundance in the rhizosphere soil were significantly higher than in the corresponding background soils for all three soil types. Comparing rhizosphere soils between soil types, bacterial diversity and abundance were significantly higher in laterite than in calcareous soil and loess, and significantly higher in calcareous soil than in loess. This ranking pattern (laterite > calcareous soil > loess) was also observed in background soils (CK3 [laterite] > CK2 [calcareous soil] > CK1 [loess]), respectively, where diversity and richness decreased from CK3 > CK2 > CK1. The influence of soil type on microbial diversity observed here aligns with previous findings (54).

Actinobacteriota, Proteobacteria, and Chloroflexi were the dominant phyla with more than 10% abundance across the three soil types. Actinobacteriota had the highest relative abundance in calcareous soil, accounting for 36.35% in the rhizosphere and 56.12% in the corresponding background soil (CK2). Proteobacteria dominated in loess (40.10%) and its background soil CK1 (46.00%), while Chloroflexi relative abundance was significantly higher in loess compared to other soil types. Actinobacteria are frequently the dominant bacterial phylum in rhizosphere soils. They possess the ability to degrade lignin and cellulose (55, 56), can produce spores to survive harsh environments, and play a role in carbonate rock weathering (57). Proteobacteria, often the most abundant bacterial group in soils, encompass diverse metabolic species, including many involved in soil nitrogen fixation (58). Chloroflexi represent a distinct phylogenetic lineage widespread in the environment, particularly thriving in low-carbon habitats (59). In the three tested soils, the abundance of Acidobacteria showed a significant negative correlation with soil pH (*r* = −0.82, *P* < 0.01). This correlation is consistent with our observations: the acidic laterite (pH = 4.48) had the highest Acidobacteria abundance (18.2%), whereas calcareous soil (pH = 6.42) had the lowest (5.7%). It is also consistent with their utilization of various carbohydrates and nitrogen sources (60) and their reported association with lower soil pH (61). This study identified soil pH and AK as the primary drivers of the bacterial community, while pH, AK, and TP were the key factors for fungi. This finding partially aligns with Tieman et al. (31), who also emphasized soil properties over host genotype. However, it contrasts with the work of Sangwanangkul et al. (32), who identified SOM as the dominant factor. This discrepancy is likely attributable to distinct edaphic contexts. The black soils in reference 32 had inherently high and less variable organic matter content, which may have amplified their role as a limiting factor. In contrast, our study utilized soils with a broader pH gradient (4.48–6.42). As pH is a master regulator of nutrient solubility and microbial physiology, it potentially overshadowed the influence of SOM, thereby highlighting pH and potassium availability as the principal drivers in our experimental context.

Additionally, we identified distinct dominant bacterial taxa enriched in each soil type: for loess, *Rhodococcus*, *Bradyrhizobium*, *Ramlibacter*, *Ralstonia*, *Lysobacter*, *Massilia*, and *Sinomonas*; for calcareous soil, *Rubrobacter* and *unclassified_o_Gaiellales*; for laterite, *Firmicutes*, *Cyanobacteria*, and *Nitrospirota*. These taxa possess ecologically relevant traits: *Firmicutes* are known for survival in harsh conditions (62) and degradation of pollutants like PCBs and petroleum hydrocarbons (63); *Nitrospirota* are crucial for nitrification and nitrogen cycling, supporting plant growth (64); *Cyanobacteria* can provide organic matter and oxygen via nitrogen fixation and photosynthesis (65). Additionally, *Sphingomonas* are known for phosphate solubilization (66), and their relative abundance increases across all soils following tomato cultivation, with the highest proportion observed in laterite treatment.

Soil microbial communities underpin ecological functions, reflected in their functional gene repertoire. Predictive functional profiling revealed six primary KEGG pathway categories, including Metabolism, Genetic Information Processing, and Environmental Information Processing, present across all soil types. Metabolism-associated genes, essential for energy acquisition, nutrient utilization (vitamins, carbohydrates), and bacterial survival, were the most abundant functional category (2). Within metabolism, carbohydrate metabolism genes regulate carbohydrate processing, while amino acid metabolism genes are linked to nitrogen cycling (67). Our analysis predicted significant differences in the relative abundance of genes associated with these specific pathways: calcareous soil showed the highest predicted abundance for carbohydrate metabolism genes, while loess soil showed the highest predicted abundance for amino acid metabolism genes.

### Characteristics of soil fungal community structures and their functions in rhizospheres of tomato grown in three soil types

Fungi constitute a vital component of soil ecosystems, serving as both pathogens and symbionts of plants and animals (68). They perform a range of critical ecological functions, including decomposition and parasitism (69). Furthermore, soil microbial diversity is recognized as a key factor contributing to soil suppressiveness against soil-borne diseases (70). In this study, following tomato cultivation, we observed significantly higher fungal diversity and richness in laterite soil compared to both calcareous soil and loess. However, no significant difference in these indices was detected between calcareous soil and loess.

The soil fungal communities across different soil types were dominated by the phyla Ascomycota and Basidiomycota, consistent with their prevalent roles in structuring soil microbial communities globally. Ascomycota encompasses many saprophytic species (71), whose growth is closely tied to nitrogen availability (72). This phylum plays a key role in degrading complex organic matter, including cellulose and lignin (73). Similarly, Basidiomycota includes highly effective decomposers of lignocellulosic material (74).

At the genus level, several fungi with significant ecological or plant-associated functions were identified. *Aspergillus* demonstrated potential for biocontrol, inhibiting pathogen mycelial growth and spore production *in vitro*, and controlling tomato disease development (75). *Trichoderma* spp. promote root growth, regulate nutrient supply and phytohormones, aiding in disease response and suppressing soil-borne pathogens (76, 77). *Penicillium* plays a key role in the rhizosphere, producing plant-beneficial compounds (e.g., soluble phosphorus, siderophores, phytohormones) and suppressing tomato anthracnose (78, 79). While often recognized as pathogens, *Fusarium* species are frequently saprophytic and play important roles in nutrient cycling and the dynamics of plant disease initiation and suppression (80). *Mortierella* contributes to decomposing complex plant litter, enhancing plant phosphorus uptake, decomposing complex carbon sources, promoting plant growth, and improving soil health (81, 82).

Our experimental results revealed significant shifts in fungal genus abundance following tomato cultivation: The relative abundance of *Aspergillus* increased substantially in the rhizosphere soil of all three soil types, with tomato ranging from 17.4% to 27.06%, compared to only 4.49% to 13.99% in background soils. *Penicillium* was significantly more abundant in calcareous soil, showing 3.37-fold and 6.97-fold higher abundance compared to loess and laterite, respectively. Furthermore, *Trichoderma* and *Fusarium* were genera associated with calcareous soil, while *Mortierella* was uniquely characteristic of laterite.

These findings suggest that the tomato root system influences the surrounding soil, leading to the enrichment of specific beneficial fungal genera. This rhizosphere enrichment may contribute to enhancing plant resistance and potentially foster a more suppressive environment against pathogens.

FUNGuild, a database enabling the identification of fungal nutritional modes and the prediction of ecological functions through comparison (83), revealed five main nutrient types across the studied soil types. Saprotrophs, which derive nutrients by decomposing dead organic matter and are typically abundant in organic-rich soils (84), were significantly enriched in calcareous soil. In contrast, loess exhibited significant enrichment of both pathotrophs and fungi with combined pathotroph-saprotroph-symbiotroph nutritional modes; pathotroph obtains nutrients by infecting and destroying living host cells (85). Laterite soil showed significant enrichment of fungi with a saprotroph-symbiotroph mode. These functional guild distributions align with physicochemical characteristics of the respective soil types.

### Conclusions

Soil type plays a determinant role in shaping the root-associated microbial community of tomato plants. Our results indicate that tomato roots selectively recruit distinct microbial assemblages across different soil types, a strategy that may enhance nutrient acquisition and modify the rhizosphere micro-environment. This selective recruitment was reflected in both functional and compositional shifts within the rhizosphere microbiome. Specifically, planting tomatoes in calcareous soil significantly increased β-glucosidase activity and MBC compared to the other soil types. Conversely, planting in laterite soil significantly enhanced acid phosphatase activity and MBP. Moreover, the rhizosphere of laterite-grown tomatoes was enriched for the bacterial genera *Bacillus, Sphingobium, Nitrospira,* and *Microvirga*, and the fungal genera *Curvularia, Mortierella, Conocybe, Humicola*, and *Roussoella*. These findings underscore the critical influence of soil type on the structure and function of tomato rhizosphere microbial communities. Understanding these dynamics can help optimize soil management practices to enhance nutrient acquisition and improve plant health in tomato production systems.

## Data Availability

The raw data for rhizosphere bacterial and fungal sequencing were deposited in the NCBI Sequence Read Archive (SRA) database under accession numbers PRJNA1297938 and PRJNA1298249, respectively.
